# Induction of Constitutive High-Level Expression of *c-Myc* in 32D Cells by Mycoplasmas is Associated with their Ability to Prevent Apoptosis and Induce Malignant Transformation

**Published:** 2006-12

**Authors:** Shimin Zhang, Shien Tsai, Shyh-Ching Lo

**Affiliations:** *Division of Molecular Pathobiology, Department of Environmental and Infectious Disease Sciences, American Registry of Pathology, Armed Forces Institute of Pathology, Washington, DC 20306, USA*

**Keywords:** apoptosis, cell cycle, malignant transformation, Myc, mycoplasmal infection, ras

## Abstract

Our previous studies showed that mycoplasmas prevented apoptosis and induced the malignant transformation of mammalian cells. Other studies indicate that *c-Myc* plays an important role in promoting apoptosis and malignant transformation of cells. To understand the role of *c-Myc* in the mycoplasma induced apoptosis prevention and malignant cell transformation, 32D cells, an IL-3 dependent cell line, were infected and transformed by different species of mycoplasmas. The expression of Myc and ras gene families, apoptosis and the cell cycle during the infection and transformation were examined. Results showed that *c-Myc* expression was significantly increased in mycoplasma transformed 32D cells. Withdrawal of IL-3 substantially decreased *c-Myc* expression and led to cell cycle arrest at the G_1_ phase followed by rapid apoptosis. Infection by *M. fermentans* or *M. penetrans* not only alleviated the sharp decrease of *c-Myc* expression, rescued 32D cells from cell-cycle arrest and prevented apoptosis in IL-3-free culture, but also induced autonomous growth of 32D cells. Although *M. hominis* and *M. salivarium* had the ability neither to prevent apoptosis nor to induce malignant transformation, they were still able to rescue the cells from cell cycle arrest. The expression of ras family did not change significantly during the infection and transformation. These results suggest that constitutive expression of *c-Myc* appears to be associated with the continuous growth and malignant transformation of 32D cells induced by *M. fermentans* and *M. penetrans*, but not with rescuing the cell cycle arrest by the mycoplasmas.

## INTRODUCTION

Mycoplasmas are the smallest microorganisms, capable of self-replication. These microorganisms commonly colonize animals and human bodies as commensals or pathogens (1). Although the mycoplasmal infections could cause certain kinds of diseases (2) and might be cofactors for acquired immune deficient syndrome (AIDS) (3), the majority of people who are infected or colonized by mycoplasmas show no clinically significant signs and symptoms. However, we believe that chronic infections by these low-virulence microorganisms might have a significant impact on host cells. It has been observed in early years that mycoplasmal infections could cause chromosomal change and transformation of mammalian cells *in vitro* (4, 5). Our recent studies have demonstrated that mycoplasmal infections significantly altered gene expression of human cells (6). Chronic infection with *M. fermentans and M. penetrans* induced malignant transformation of C3H cells, a mouse embryonic cell line with low inherent spontaneous transformation (7) and 32D cells (8).

32D cells, a mouse multi-potential hematopoietic progenitor cell line, are strictly dependent on interlukin-3 (IL-3) for their growth (9). Withdrawal of IL-3 from 32D cell culture leads to rapid programmed cell death (apoptosis) (10, 11). Infections with *M. fermentans or M. penetrans* prevent the apoptosis and long-term infections with these mycoplasmas induces malignant transformation of the cells (8). We have successfully established transformed 32D cell lines by chronic infections with *M. fermentans and M. penetrans.* These cell lines can form tumors after injected into nude mice (8). We believe that the malignant transformation of 32D cells induced by mycoplasmal infections is accomplished through two separate avenues. In the first avenue, mycoplasmas rapidly activate an anti-apoptotic pathway(s) such as NFκB pathway and support continuous cell growth in the IL-3 free condition (8). In the second avenue, mycoplasmas induce malignant transformation that requires infection of live organisms with a latent period and causes the infidelity of genomic transmission in cell division. Despite these interesting findings, the mechanism of the malignant transformation induced by the mycoplasmas is still not clear.

The *c-Myc* proto-oncogene was first described in 1982 as the cellular homologue to the transforming sequences of the avian myelocytomatosis retrovirus (12). Mutated *c-Myc* oncoproteins induce malignant cell transformation. Not only did Mutated *c-Myc* oncoproteins induce cell transformation, augmented expression of normal *c-myc* was also sufficient for cotransformation of rat embryo cells with a mutant ras gene (13). It was soon found that activated oncogenic *c-Myc* is a key transforming factor in the etiology of human Burkitt’s lymphoma (14). In fact, over-expression of *c-Myc* is implicated in many oncogenic processes of both naturally occurred malignant tumors and *in vitro* induced malignant cells (15, 16) (17, 18). Paradoxically, *c-Myc* also induces apoptosis (19, 20) and the enforced expression of high level of *c-Myc* accelerated apoptosis of 32D cells in IL-3-free cultures (10), which is a key cancer-preventing mechanism. Transformation of 32D cells by mycoplasmal infections involves the prevention of cell apoptosis (8). Since overexpression of *c-Myc* was observed in our previous mycoplasma induced malignant transformation CH3 cell model (21), it will be very interesting to know the role of *c-Myc* in both apoptotic prevention and malignant transformation of mycoplasma infected 32D cells. In this study, the expression of myc and ras oncogene families in 32D cells was studied during mycoplasmal infection and subsequent transformation. Growth, cell cycle and apoptosis of affected cells were monitored. A linkage between *c-Myc* expression and prevention of apoptosis as well as induction of malignant transformation by mycoplasmas was explored.

## MATERIALS AND METHODS

### Mycoplasmal infection of 32D cells

32D cells were cultured in RPMI 1640 medium containing 10% fetal bovine serum and 8% WEHI cell conditioned medium (as IL-3 source). 32D/GTU/c, 32D/MI/c and 32D/PG/c are IL-3 independent 32D cell lines established by transforming 32D cells with *M. penetrans* GTU-54 strain, *M. fermentans* incognitus (MI) and PG18 strains, respectively (8). Mycoplasmas in these transformed cell lines have been eradicated by antibiotic treatment. They are able to grow in both IL-3 containing and IL-3 free medium. Cell viability was determined by Trypan blue exclusion assay (22).

*M. fermentans* (PG 18), M. salivarium and M. hominis PG21T were obtained from National Institute of Health, Bethesda, Maryland; *M. penetrans* (GTU-54) and *M. fermentans* MI were isolated from a patient with AIDS in this laboratory (23, 24). The mycoplasmas were cultured in SP4 medium containing 18% fetal bovine serum, 100 units per ml of penicillin and 500 units per ml of polymyxin (23). Mycoplasmas in exponential growth judged by the color change of the culture medium were aliquoted and stored at -70°C. Quantification of mycoplasmas was performed by measuring the color changing unit (CCU) or colony forming unit (CFU) as described in our previous study (6). All mycoplasmal cultures were quantified by at least one of the above methods.

To infect 32D cells, 6 ml of mycoplasmal stock was added into 100 ml of cell culture at 0.5 × 10^6^ to 1 × 10^6^ cells/ml in a 175 cm^2^ flask with or without IL-3 supplementation. The same volume of SP4 medium was added into control flasks. Titers of the mycoplasmal stocks used in this study were about 10^7^ CCU.

### RNase Protection Assay

Total RNA was prepared from the cells using TRIzol reagent (GIBCO BRL, Gaithersburg, MD) according to the manufacturer’s instruction (25). RNAs were dissolved in DEPC (Diethyl pyrocarbonate) treated H2O. The quantity and purity of the RNA were determined by measuring the absorbance at 260 nm and the 260/280 absorbance ratio on a spectrophotometer and by electrophoresis on 1.2% agarose gel using formaldehyde-MOPS (3-[N-Morpholino]propanesulfonic acid) buffer.

mRNAs of myc and ras gene families were analyzed by the RNase protection assay (RPA) using RiboQuant multi-probe kit (PharMingen, San Diego, CA) following instructions of the manufacturer. In brief, 32P-labeled specific anti-sense RNA probes were synthesized by *in vitro* transcription from multiple DNA template sets (m-Myc and m-Ras, from PharMingen), which include two housekeeping genes, L32 and GAPDH (glyceraldehyde 3-phosphate dehydrogenase) as loading controls. An equal amount of total RNA (20 μl for each sample) was hybridized overnight to the 32P-labeled RNA probes. Unhybridized RNAs and RNA probes were digested with RNases A and T1. The protected mRNAs and probes were precipitated with ethanol and resolved on a 6% denaturing urea polyacrylamide gel. Undigested 32P-labeled probes serve as molecular size markers. The gel was dried and exposed to X-ray film as well as to a phosphoimage screen. The expression of mRNA transcript of a specific gene was quantified on a Storm 860 phosphoimage scanner followed by the analysis with software ImageQuant (Molecular Dynamics, Sunnyvale, CA).

### Apoptosis assay

Cell apoptosis was assessed by measuring nucleosome levels in cell lysates using a nucleosome ELISA kit (Oncogene Research Products, Cambridge, MA). Briefly, Cells from 5 to 10 ml of culture were counted, harvested by centrifugation and lysed in 1 ml of lysis buffer. The lysates were diluted with the lysis buffer to equivalent 10^6^ cells/ml and frozen at -20°C for at least 24 h before the assay. To measure nucleosome, cell lysates were added to a 96-well enzyme-linked immunosorbent assay (ELISA) plate that has been coated with anti-nucleosome antibodies. The absorbed nucleosomes on the plate were detected by a biotinylated detector antibody and streptavidin-peroxidase conjugate, and quantified on an ELISA reader.

### Determination of cell cycle

Cells were collected and washed once with phosphate buffer saline (PBS) by centrifugation, and then fixed in 50% ethanol. The fixed cells were stored at 4°C until analyzed by flow cytometry. To determine cell cycle distributions, the fixed cells in 50% ethanol were washed with PBS once. To stain cells, about 10^6^ cells were resuspended in 1 ml of 0.1M Tris-HCl, pH7.2 containing 0.07M NaCl, to which equal volume of 10μM 4',6-diamidino-2-phenylindole (DAPI) in 800mM Na_2_HPO_4_ was added. The cell suspension was incubated at 4°C overnight. Fluorescence of DAPI stained cells was measured on a flow cytometer. The percentages of cells in G_0_/G_1_, S and G_2_/M phases were determined.

## RESULTS

### Mycoplasma-transformed 32D cells constitutively express high levels of *c-Myc*

**Figure 1:**
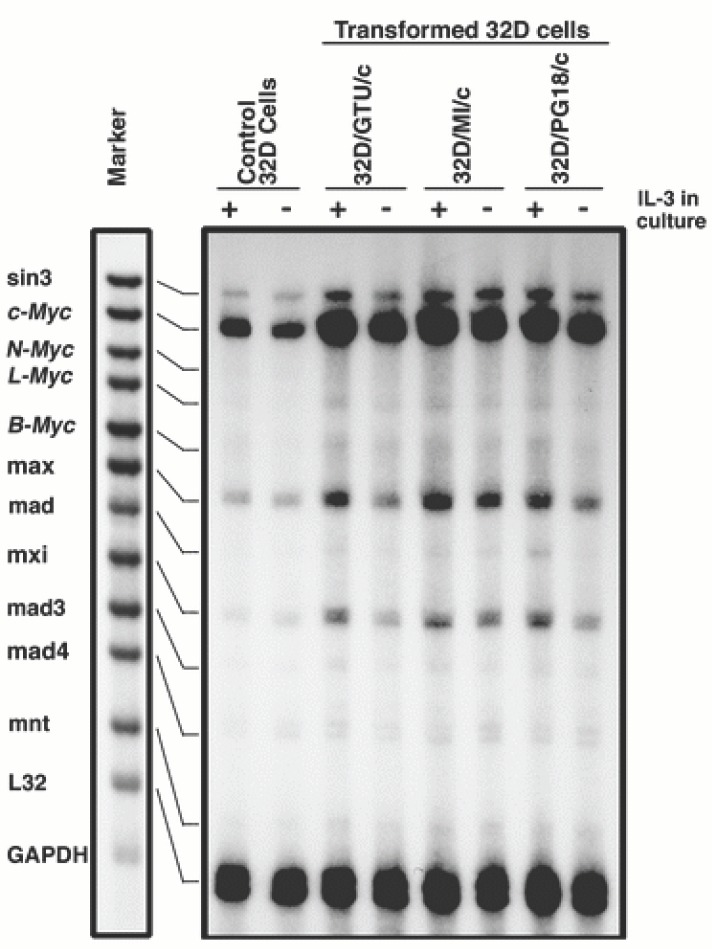
Expression of Myc oncogene family in mycoplasma-transformed and control 32D cells. Mycoplasma-transformed and control 32D cells cultured in RPMI 1640 medium containing 10% FBS and IL-3 at least for 3 weeks were harvested by centrifugation. The cells were transferred to fresh culture medium with (+) or without IL-3 (-) and cultured for 36 hours The expression of myc oncogene family (mRNA) in the cells was determined by the RPA.

The expression (mRNA) of myc oncogene family (*sin 3, c-Myc, L-Myc, L-Myc, B-myc, Max, mad, mxi, mad3 mad4 and mnt*) in mycoplasma-transformed 32D cell lines 32D/GTU/c, 32D/MI/c and 32D/PG/c was examined by using the multiple probe RPA. Parental 32D cells were used as control. In the presence of IL-3, control 32D cells expressed a relatively high level of *c-Myc* and moderate level of sin3 and Max as well as a low level of mxi, mad4 and mnt. The expression of B-myc, L-Myc, N-Myc, mad, mad3 and mad4 was not detected in these cells (Fig. 1). Thirty-six hours after withdrawal of IL-3 from cell cultures, the expression of *c-Myc* decreased by 50 to 80%. However, the expression of other members in the myc family was little changed. In the mycoplasma-transformed 32D cells, *c-Myc* mRNA was also decreased following IL-3 withdrawal though the magnitude of the decrease was smaller than that in the control. However, the expression level of *c-Myc* mRNA in the mycoplasma-transformed cells was still 1.5-3 times higher than that in control 32D cells. In addition, the levels of sin3, Max and mxi mRNA also decreased in the mycoplasma transformed 32D cells following IL-3 withdrawal (Fig. 1).

**Figure 2:**
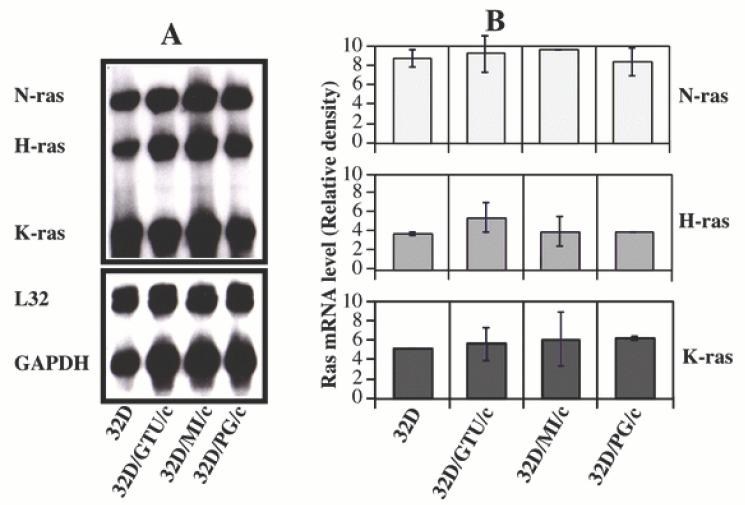
Expression of ras oncogene family in mycoplasma-transformed and control 32D cells. The expression of ras genes (Panel A, autoradiograph) was determined as described in Fig. 1 except for using the ras gene template set. Panel B. is the average of 3 independent experiments quantified by the phosphoimage analysis.

At the same time, we examined the expression of *H-ras, K-ras and N-ras* oncogenes by the same method (RPA). Results indicated that 32D cells expressed substantial amount of ras mRNAs. However, the expression level was not significantly increased in mycoplasma-transformed 32D cells (Fig. 2). Withdrawal of IL-3 from cell cultures did not significantly affect *ras* gene expression both in the mycoplasma transformed 32D cells and their control cells.

### Continuous growth of 32D cells following IL-3 withdrawal and mycoplasmal infection was associated with preventing sharp decrease of *c-Myc* expression in the cells

**Figure 3:**
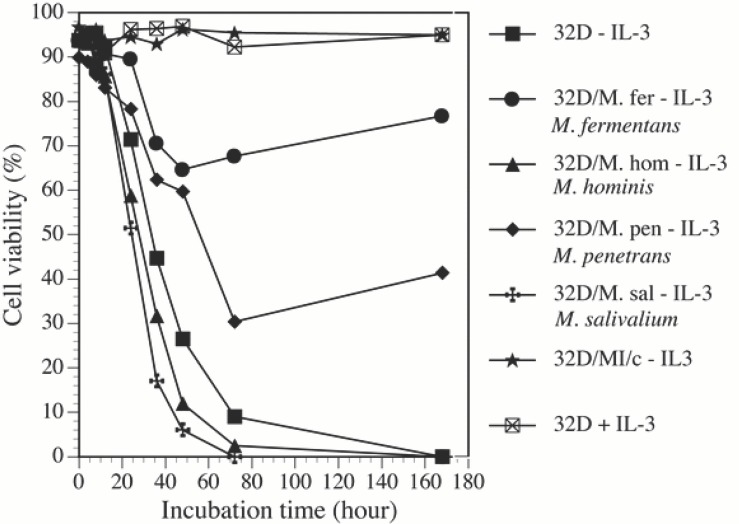
Effect of mycoplasmas on cell growth. Mycoplasma-transformed and control 32D cells cultured in RPMI 1640 medium containing 10% FBS and IL-3 at least for 3 weeks were harvested by centrifugation and then transferred into fresh medium with (+) or without IL-3 (-) immediately followed by adding mycoplasmal stocks into the cultures. The same amount of SP4 medium was added into non infected control culture. The viability of the cells at set time points was examined by the trypan blue exclusion assay. The results represent 3 similar experiments.

**Figure 4:**
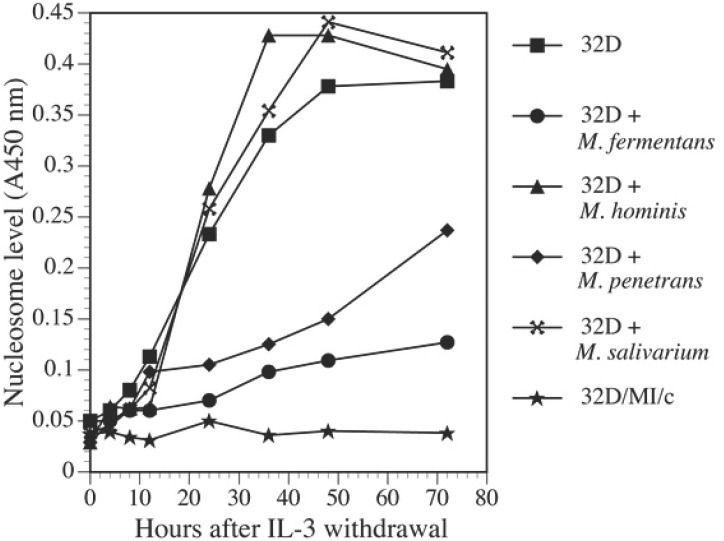
*M. fermentans* and *M. penetrans* prevented apoptosis of 32D cells following IL-3 withdrawal. Cells were harvested and infected with mycoplasmas in IL-3 free cultures as described in Fig. 3. Nucleosome level in cells at different time points was measured by the ELISA assay. The results are representative of 3 separate experiments.

Mycoplasmal infections supported continuous growth of 32D cells in IL-3 free cultures. In this study, we showed that different species of mycoplasmas had different effects on the growth of IL-3 dependent 32D cells in IL-3 deprived cultures (Fig. 3). Withdrawal of IL-3 from cultures caused rapid cell death in noninfected control cell cultures. About 50% of the control cells survived 36 h following IL-3 withdrawal. Only around 10% remained alive on day 3. Almost all control cells died within 7 days. In *M. fermentans* infected cultures, although some cells died during the first week of infection many cells continued to proliferate. Cell viability in the infected cultures was about 70% and 55% respectively after 36 h and 72 h infection by the mycoplasma. The viability gradually improved 5 days later. *M. penetrans* could also prevent 32D cell death due to IL-3 withdrawal as *M. fermentans* did, but it was less effective than *M. fermentans*. However, *M. salivarium* and *M. hominis* did not have any protective effect on 32D cells following IL-3 withdrawal. By using nucleosome ELISA assay, we showed that supporting 32D cell continuous growth by *M. fermentans* and *M. penetrans* was accompanied by preventing these cells from apoptosis in IL-3 free cultures (Fig. 4). Similar to the results obtained from the viability assay, *M. fermentans* was near twice as potent as *M. penetrans* in preventing 32D cells from the programmed cell death following IL-3 withdrawal. Apoptotic cells in IL-3 free cultures infected with *M. fermentans* or *M. penetrans* were about 70% and 50% respectively less than those found in non-infected control cultures 24 h following the infection. However, *M. hominis* and *M. salivarium* did not have the ability to protect 32D cells from the apoptosis due to IL-3 deprivation (Fig. 4).

**Figure 5:**
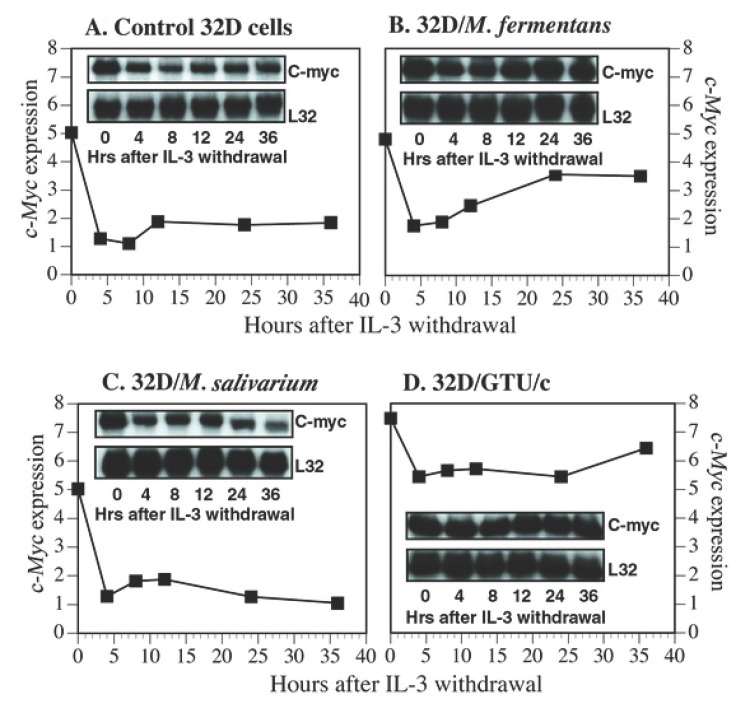
Kinetics of *c-Myc* expression in mycoplasma infected, transformed and control 32D cells following IL-3 withdrawal. Exponentially growing cells were transferred to IL-3-free cultures and infected with M. fermentans (panel B) or M. salivarium (panel C). Non-infected 32D cells (panel A) and M. penetrans transformed 32D cells (panel D) were also tested. The expression of *c-Myc* in the cells was determined by the RPA (inlets). Results were quantified by the phosphoimage technique and normalized by the expression of a house-keeping gene L32.

*c-Myc* promotes apoptosis (20). Enforced *c-Myc* expression accelerates 32D cell apoptosis process (10). Our results shown above have revealed that the expression of *c-Myc* in 32D cells is significantly increased following transformation by *M. fermentans* and *M. penetrans*. These two mycoplasmas can effectively prevent 32D cells form apoptosis, which, we believe, is an early stage of the mycoplasma induced transformation process. It is not clear if acute mycoplasmal infections also have an impact on the *c-Myc* expression in this stage. To address this question, short-term mycoplasma infected 32D cells and noninfected control cells were examined for the *c-Myc* expression. Exponentially growing 32D cells were transferred into IL-3 free medium. At the same time, the cells were infected with 4 different species of mycoplasmas, *M. fermentans, M. penetrans, M. salivarium* and *M. hominis*. Total RNA was prepared from the infected and noninfected control cells. The expression of *myc* and *ras* gene families in these cells at 0 h, 4 h, 8 h, 12 h, 20 h and 36 h was determined by the multiple-probe RPA. Results showed that the expression of *c-Myc* mRNA in mycoplasma-infected and control 32D cells rapidly decreased within 4 h following IL-3 withdrawal. In control cells, the *c-Myc* mRNA level remained low until cells died (Fig. 5A). In *M. fermentans* or *M. penetrans* infected 32D cells, after a short period of decrease, the *c-Myc* expression bounced back to and maintained at a relative high level (Fig. 5B). In contrast, *M. salivarium* and *M. hominis* could not support the growth of 32D cells in IL-3 deprived cultures. The expression of *c-Myc* in these cells was similar to that in control cells (Fig. 5C). Mycoplasma-transformed 32D cells that had grown in IL-3 containing cultures for more than 2 weeks also experienced a temporary slight decrease of *c-Myc* mRNA short after IL-3 withdrawal. However, the expression level returned to close to its original high level within 36 h (Fig. 5D). The results indicated that the expression of *c-Myc* was strongly correlated with supporting continuous cell growth by mycoplasmas and IL-3 still had some influence on *c-Myc* expression in mycoplasma-transformed 32D cells. Infections by the transforming mycoplasmas upregulated *c-Myc* expression and partially compensated the decrease of *c-Myc* expression following IL-3 withdrawal. On the other hand, the expression of *ras* oncogene family was independent of IL-3 regulation and was not significantly affected by the mycoplasmal infection.

### Mycoplasmal infections rescued 32D cells from cell-cycle arrest in the absence of IL-3

**Figure 6:**
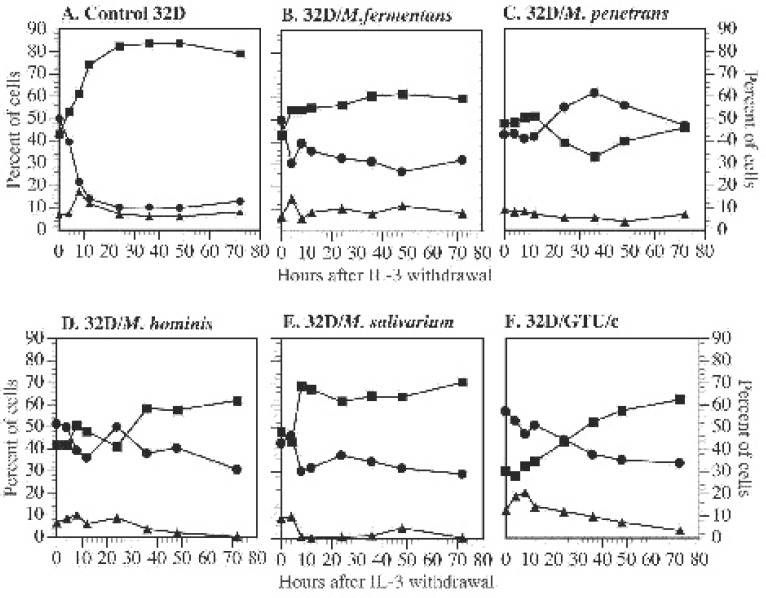
Mycoplasmal infections rescued 32D cells from cell-cycle arrest at G_1_ phase in the absence of IL-3. Cells were treated in the same way as described in Fig. 5. These cells included control 32D cells (panel A), 32D cells newly infected M. fermentans (panel B), M. penetrans (panel C), M. hominis (panel D) or M. salivarium (panel E), and M. penetrans transformed cells (panel F). The cells were harvested and prepared at set time points for the cell cycle analysis by flow cytometry. ^■^G_1_ phase: ●; S phase: ▲; G_2_/M phase.

Withdrawal of IL-3 leads to apoptosis of 32D cells within a few days. Within the first 12 h, the cells quickly respond to IL-3 withdrawal by arresting the cell cycle at the G_1_ phase.(10, 11) Enforced expression of the high level of exogenous *c-Myc* rescued 32D cells from the cell cycle arrest. However, overexpression of exogenous *c-Myc* could not change the apoptotic fate of 32D cells due to IL-3 withdrawal, but could enhance the apoptotic process (10). The question is whether mycoplasmal infections and increased expression of endogenous *c-Myc* has any impact on the cell cycle of 32D cells. To answer this question, we infected 32D cells with mycoplasmas in IL-3 free cultures. Cell cycle distributions of the infected cells, noninfected control 32D cells and three mycoplasma transformed 32D cell lines were examined by the flow cytometry. As expected, withdrawal of IL-3 caused control cells to arrest at the G_1_ phase. The cells in the G_1_ phase rapidly accumulated and reached a plateau about 24 h after IL-3 withdrawal (Fig. 6A). These cells died in a few days following this G_1_ phase arrest. However, 32D cells infected with M. fermentans, M. penetrans, M. hominis and M. salivarium did not stop at the G_1_ phase following IL-3 withdrawal (Fig.6B, C, D and E). All four species of mycoplasmas tested could rescue 32D cells from cell cycle arrest at the G_1_ phase in IL-3 deprived cultures and, drove the cells to pass the G_1_ phase checkpoint and to progress into the S phase. The promotion of this cell cycle progression by mycoplasmas is independent of their ability to prevent apoptosis of 32D cells in IL-3 deprived cultures. However, 32D cells infected with M. hominis and M. salivarium had fewer G2/M phase cells than those infected with *M. fermentans* and *M. penetrans*. These results suggest that although M. hominis and M. salivarium could drive the cells to pass the G_1_ phase checkpoint they were less efficient to drive cells to pass the S phase checkpoint. On the other hand, IL-3 independent, mycoplasma-transformed 32D cells continuously proliferated in the absence of IL-3 or mycoplasmas (Fig. 6F). Results described here and in the above section indicate that there is no close relationship between the expression level of endogenous *c-Myc* in 32D cells and cell cycle distribution during mycoplasmal infection and cell transformation.

## DISCUSSION

Our previous study has revealed that the expression of *c-Myc* was dramatically increased in mycoplasma transformed C3H cells (21). In this study, we demonstrated that 32D cells expressed relatively high levels of *c-Myc* and Max transcripts. Withdrawal of IL-3 led to rapid decrease of *c-Myc* expression, but did not obviously affect Max expression. However, mycoplasma-transformed 32D cells constitutively expressed high levels of *c-Myc* in the presence or in the absence of IL-3. These results imply that *c-Myc* has a critical role in mycoplasma induced malignant transformation of 32D cells. These results are consistent with previous studies on the role of *c-Myc* in cell malignant transformation (26-28). The increased *c-Myc* oncoproteins in mycoplasma transformed cells can form dimers with its partners, Max or other related proteins, which then bind to the specific DNA sequence (CACA/GTG), activate transcription and regulate cell biological functions. Although early studies indicated that *c-Myc* and *N-myc* were equally potent in transforming mammalian cells and L-myc also had weak transformation ability (29) we were unable to detect the expression of N-myc and L-myc in 32D cells before and after the transformation. Apparently, these two oncogenes are not associated with mycoplasma induced malignant transformation.

We have noticed that 32D cells expressed substantial amount of N-ras, H-ras and K-ras. Although there was no significant increase in the expression of the ras oncogenes in mycoplasma transformed 32D cells, the role of the *ras* gene family in this transformation process could not be completely excluded. .*c-Myc* collaborates with activated ras oncogenes in transforming primary embryonic rat fibroblasts *in vitro* (30). Our previous study also demonstrated the increased expression of *c-Myc* and H-ras in mycoplasma transformed C3H cells (21). Constantly expressed *ras* oncoproteins in 32D cells might be sufficient to collaborate with increased *c-Myc* to promote malignant transformation of 32D cells during chronic mycoplasmal infection. The true role of *c-Myc* in mycoplasma induced malignant transformation might be confirmed by using *c-Myc* small interfering (si) RNA or *c-Myc* repressors, such as FUSE-binding protein-interacting repressor (FIR) (31, 32).

Not only can *c-Myc* promote proliferation and transformation of mammalian cells, but it is also involved in apoptosis. Rat-1 fibroblasts with the high level of *c-Myc* proteins are more prone to programmed cell death upon serum deprivation (33). Enforced expression of high levels of *c-Myc* in 32D cells accelerated apoptotic process in IL-3 free culture (10, 34). Abnormal or overexpression of *c-Myc* alone in cells induced apoptosis by inducing a p53-dependent cell death pathway, hence, protecting the organism from lethal neoplastic changes (35, 36). On the other hand, results of our studies and others (10) have shown that *c-Myc* in untreated 32D cells rapidly decreased within 4 h following IL-3 withdrawal, followed by rapid cell apoptosis. Furthermore, when 32D cells were infected with mycoplasmas, *M. fermentans* and *M. penetrans* not only supported continuous growth of 32D cells in IL-3 free cultures, but also prevented the sharp decrease of *c-Myc* expression following IL-3 withdrawal. M. hominis and M. salivarium that did not support continuous cell growth in the same IL-3 free culture condition, could not maintain constitutive expression of high levels of *c-Myc*. These results suggest that *c-Myc* in 32D cells might act as a survival factor instead of an apoptotic inducer and decrease of endogenous *c-Myc* expression is associated with apoptosis. The role of endogenous *c-Myc* seems to be completely different from that of over-expressed exogenous *c-Myc* in supporting the growth of 32D cells. In fact, co-expression of *c-Myc* and Bcl-xL, a apoptosis repressor and oncogene, not only suppress *c-Myc*-induced apoptosis but also expose multiple oncogenic properties of *c-Myc* and triggers carcinogenic progression (35, 37). M. fermentans and M. penetrans, but not M. hominis nor M. salivarium, increased the expression of Bcl-xL in infected 32D cells (Zhang S, et al. unpublished data). Collaborative actions of *c-Myc* and Bcl-xL might be the mechanism, through which mycoplasmas prevent apoptosis of 32D cells in IL-3 free condition and induce malignant transformation of the cells.

*c-Myc* anti-sense transcripts inhibit G_1_ progression by reducing expression of endogenous *c-Myc* in Friend murine erythroleukemia cells (F-MEL) (38). Withdrawal of IL-3 causes cell cycle arrest of 32D cells at the G_1_ phase and over expression of exogenous *c-Myc* could rescue 32D cells from the cell cycle arrest (10, 39). These evidences indicate that *c-Myc* plays an important role in cell cycle progression (39, 40). The results from this study showed that mycoplasma mediated G_1_ progression of 32D cells was independent of the expression of endogenous *c-Myc*. Only *M. fermentans* and *M. penetrans* among four species of mycoplasmas tested can prevent the decrease of *c-Myc* expression in 32D cells following IL-3 withdrawal. However, all four species of mycoplasmas tested can also prevent the G_1_ phase arrest of 32D cells caused by IL-3 withdrawal. The results indicate that the cell cycle progression induced by mycoplasmas might go through other pathway(s) rather than the *c-Myc* pathway. Since *M. hominis* and *M. salivarium* failed to rescue the IL-3-free 32D cells from dying of apoptosis, c-Myc could be critical in releasing cell arrests at other check points of the cell cycle.

Generally speaking, withdrawal of growth factors can cause cell cycle arrest at the G1 phase by affecting the G1 phase checkpoint. DNA damage may provent cells from entering the S phase and the G2 phase by affecting DNA replication checkpoint (41). The G_1_ phase arrest caused by negative growth factors is easily overcome by supplying corresponding growth factors or by adding a growth factor inducer. When the cell cycle is stopped by DNA damage, DNA repair may restore the cell cycle or the cells undergo apoptosis. Our previous studies have revealed that mycoplasmas and mycoplasmal associated membrane proteins (LAMPs) induce the expression of many cytokines (6). Some of these cytokines can support cell growth (42). Therefore, it is not surprising that mycoplasmas can prevent cell cycle arrest at the G1 phase following growth factor withdrawal.
